# Evaluating transdisciplinary methods: a new scale for measuring knowledge integration

**DOI:** 10.1057/s41599-025-05634-w

**Published:** 2025-08-26

**Authors:** Cornelia Fischer, Katharina Gugerell, Ursula Laa, Jannik Jacobsen, Marianne Penker

**Affiliations:** 1 Department of Economics and Social Sciences, BOKU University, Vienna, Austria; 2 Department of Landscape, Water and Infrastructure, BOKU University, Vienna, Austria; 3 University of Johannesburg, Johannesburg, South Africa; 4 Department of Natural Sciences and Sustainable Resources, BOKU University, Vienna, Austria; 5 University of Vienna, Vienna, Austria

**Keywords:** Science, technology and society, Environmental studies

## Abstract

Transdisciplinary research presents a promising approach to addressing complex societal challenges by integrating scientific and experiential knowledge in joint learning processes. Transdisciplinary methods are crucial for supporting knowledge integration by enabling actors from within and outside academia to evaluate their expertise, share insights, and co-create innovative solutions. Despite growing interest in transdisciplinary methods, their effectiveness remains under-researched, mainly due to a lack of standardized instruments to measure their contribution to knowledge integration. This gap is particularly significant given that the usefulness of transdisciplinary methods depends on knowledge integration as a multidimensional, iterative process that fosters learning without predetermined outcomes. In this study, a novel scale was developed and tested empirically to assess the contribution of transdisciplinary methods to knowledge integration. The scale development process involved a systematic review of 48 literature sources, which synthesized over 300 statements into 34 items. These items were tested in workshops with 71 participants using two different transdisciplinary methods: transdisciplinary scenario building and serious game development. The participants represented expertise from three academic disciplines and experiential knowledge from the dairy and meat supply chains as well as game development. Exploratory factor analysis revealed two distinct dimensions of knowledge integration: a socio-emotional factor and a cognitive-communicative factor. This finding resulted in a refined 25-item scale. The scale was then employed to compare the two transdisciplinary methods, thereby providing an instrument for comparative analysis of their respective contributions to knowledge integration. The article also underscores the scale’s limitations and offers recommendations for future scale refinement. By improving the methodological basis for measuring transdisciplinary methods, this research contributes to the ongoing improvement of transdisciplinary research.

## Introduction

Transdisciplinary (td) research is increasingly seen as a promising approach to address complex societal challenges and to generate new knowledge by transcending disciplinary boundaries and integrating the knowledge of scientific actors and societal actors (Andrews et al. 2024; Bammer et al. 2020; Kalmár and Stenfert 2020; Lux et al. 2024; OECD 2020; Pohl 2024). Td research extends beyond science to tackle complex, high-stakes issues (Hoffmann-Riem 2008; OECD 2020). By integrating diverse perspectives, it fosters innovative, holistic solutions, generating both scientific insights and societal benefits (de Rezende Alvares and de Sá Freire 2022; Bergmann et al. 2010; Hoffmann-Riem 2008; Madni 2007). As a complementary approach, it enhances but does not replace disciplinary research (OECD 2020; Pärli 2023).

Knowledge integration, while a central mechanism in td research, serves a broader purpose. That purpose is to support the overarching goals of enhancing the relevance, credibility, legitimacy, and ultimately the effectiveness of research in addressing complex societal challenges. As such, the impact of td research is closely linked to the quality and design of knowledge integration employed (Lux et al. 2024). In the context of our research, the process of knowledge integration describes an iterative and reciprocal exchange between the scientific and societal actors involved, characterized by a mutual learning and dialog (Jahn and Keil 2015; Misra et al. 2024; Pohl et al. 2021) and encompassing all forms and combinations of scientific and societal knowledge and information (Misra et al. 2024; Pohl et al. 2021; Scholz et al. 2024a). It is dynamic and multifaceted, requiring careful management of different perspectives, continuous reflection, and adaptation strategies (Gugerell et al. 2023; Karrasch et al. 2022; Lux et al. 2024; Pohl et al. 2021), along with methods that help to bridge different knowledge systems (Hoffmann et al. 2017; Özerol et al. 2018). Empirical studies on the application and evaluation of td methods (TdM) remain scarce (Misra et al. 2024), while the absence of evaluation mechanisms limits their effectiveness and real-world impact (Cockburn 2022; Gugerell et al. 2023; Restrepo et al. 2018).

Despite the diversity of td research in terms of objectives and actors involved raises doubts about the feasibility of a universal model of knowledge integration (Misra et al. 2024), numerous authors refer to models that consider different dimensions of integration to enhance comprehension of the requirements for knowledge integration in td research (e.g., Bergmann et al. 2010; Jahn et al. 2012; Mitchell and Ross 2016; Peukert and Vilsmaier 2021; Pohl et al. 2021). A dimension groups specific aspects of the td process of knowledge integration (Bergmann et al. 2010; Lux et al. 2024; Pohl et al. 2021). In the field of td research, these dimensions encompass knowledge synthesis (cognitive-epistemic dimension), collaboration and interaction (social-organizational dimension), common language and discursive practice (communicative dimension), as well as respect and trust building (emotional dimension) (Bergmann et al. 2010; Boix Mansilla et al. 2016; Godemann 2008; Gugerell et al. 2023; Jahn et al. 2012; Lux et al. 2024; Mitchell and Ross 2016; Peukert and Vilsmaier 2021; Pohl et al. 2021).

In the present study, we conceptualize TdM as purposefully selected “tools to collect and organize knowledge systematically” (Minna et al. 2023) that match the specific objectives of td research and support and facilitate knowledge integration (Bammer 2008a; Bammer et al. 2020; Defila and Di Giulio 2019; Kalmár and Stenfert 2020; Lang et al. 2012; Lam et al. 2021; Minna et al. 2023; Pohl et al. 2021; Studer and Pohl 2023). TdM enables participants to identify and evaluate their own knowledge and expertise, gain new insights, and integrate knowledge from scientific and societal actors (Bergmann et al. 2010; Defila and Di Giulio 2019; Minna et al. 2023; Pohl et al. 2021; Studer and Pohl 2023; Theiler et al. 2019). Despite the growing interest in the changing roles of scientists, methodological choices and practices receive little attention in academic discourses, and there is little conscious selection of TdM (Bammer 2024; Minna et al. 2023). Td research needs to evaluate existing methods and determine whether new TdMs are needed to design research processes and support knowledge integration as a multidimensional process (Bergmann et al. 2010). The suitability of TdM depends on different aspects, including the complexity of the research question, the balance between empirical and subjective aspects (cognitive-epistemic dimension), the participants in the research dialog (communicative dimension), power dynamics (social-organizational dimension), personal interaction (emotional dimension), and the purpose of the study (Bergmann et al. 2010; McDonald et al. 2009). Documentation and evaluation of TdM are critical to understanding the elements that lead to successful research outcomes (Bammer 2013; Bergmann et al. 2010; ITD Alliance Working Group on Toolkits and Methods 2024; Laursen et al. 2024; McDonald et al. 2009; Pohl et al. 2021; Studer and Pohl 2023).

Operationalizing the theoretical dimensions of knowledge integration is regarded as difficult (Peukert and Vilsmaier 2021), and current approaches may not adequately address all dimensions of integration, favoring certain aspects over others (Bammer 2008b; Pohl et al. 2021). Since different dimensions of integration indicate the key elements for knowledge integration in td research, the development of a scale that incorporates these dimensions could serve as a useful evaluation tool. This would enable the evaluation of the integration performance of TdM, foster a deeper understanding of the underlying relationships, and provide an opportunity to gain deeper insights into effective methods and approaches. Due to the absence of an appropriate evaluation tool for TdM and its potential applications, this paper addresses the following research questions: (a) Which items can be used for evaluating the contribution of TdM to knowledge integration? (b) How can TdM be evaluated using a scale of knowledge integration?

The study aims to empirically develop and test a scale to measure the contribution of TdM to different dimensions of knowledge integration. This article describes the process of scale development and how the scale was tested to evaluate two methods to understand if scenario creation or serious game development scores better in terms of their contribution to the various dimensions of knowledge integration. Systematically analyzing the strengths and weaknesses of TdM across different dimensions of knowledge integration is essential for evaluating and guiding knowledge integration in td research. This facilitates optimized method combinations, enhances methodological innovation, and makes progress measurable. Incorporating evaluation insights into existing toolkits would allow for more conscious selection and targeted and effective use of TdM in td research. This is also important for accompanying td research, as it strengthens the knowledge integration performance of td research and contributes to optimizing methodological decisions, thereby increasing the societal and scientific relevance of td research.

The aim of developing a scale to assess knowledge integration is not to cover the entire field of td research, but to provide a focused, empirically based instrument to evaluate a central process within this field. Rather than measuring td outcomes, the scale is intended to complement existing assessment tools by considering the contribution of TdM to the various dimensions of knowledge integration.

The paper is organized as follows: The “Knowledge integration and its dimensions” section provides an overview of the current state of knowledge integration and the importance of TdM in td research. This is followed by the methodological approach and the scale development process, and the application of the scale to two TdM. The “Results” section presents our findings, which are discussed in the “Discussion” section. Finally, we draw general conclusions. This article discusses the results and limitations of the exploratory study, possible applications, and aspects contributing to the further scale development.

## Knowledge integration and its dimensions

Knowledge integration describes a social, emotional, communicative, or cognitive process in which different knowledge systems from different academic disciplines and societal fields converge (Lux et al. 2024). Transdisciplinarity emphasizes knowledge integration across disciplinary boundaries with non-academic actors to address real-world problems (Di Giulio and Defila 2024, Godemann 2008; Mobjörk 2010). Integrating scientific and experiential knowledge – two complementary epistemologies – is essential to developing a nuanced understanding of the mechanisms and conditions driving sustainability transitions. Combining these knowledge systems, particularly in contexts of high uncertainty and significant societal stakes, yields a more comprehensive analysis of transition dynamics, enabling more effective problem solving and decision making. Scientific evidence on the drivers and mechanisms of change is complemented by the real-life experiences of societal actors. Knowledge integration can result in different types of knowledge, including systems knowledge (understanding existing structures and processes), target knowledge (desired goals), and transformation knowledge (identifying pathways to achieve goals), all considered in an integrated manner (Hirsch Hadorn et al. 2008).

By exchanging perspectives, the involved actors reshape their mental models by linking data, theories, norms, values, and viewpoints in a meaningful way (Burger and Kamber 2003; Defila et al. 2006; Godemann 2008; Jahn et al. 2012; Pohl et al. 2021; Strasser et al. 2014). Thus, knowledge integration involves developing relationships between previously unrelated elements (knowledge, data, theories, norms, values, or views of individual participants) (Pohl et al. 2021). In td research, different conceptual approaches, methods, and applications are combined. This process involves mutual critique and reflection (Bammer et al. 2020; Hinrichs et al. 2017; Jahn et al. 2012; Kalmár and Stenfert 2020; Misra et al. 2024). Addressing challenges and fostering collaborative practices are essential for successful knowledge integration (Gugerell et al. 2023; Karrasch et al. 2022). Improving td collaboration includes fostering open communication, ensuring equal participation, and continuously reflecting on the integration process to adapt to evolving challenges (Pohl et al. 2021; Godemann 2008). Since objective criteria for evaluating knowledge integration are often lacking, reflective assessments by the involved actors become particularly important (Defila and Di Giulio 2015). As a collective and interactive process, knowledge integration depends on diverse perspectives and mutual understanding. Beyond integrating existing knowledge, it is also important to generate new knowledge to evaluate its contribution to scientific and societal progress (Jahn et al. 2012) and to respond consciously and reflectively to remaining knowledge gaps or uncertainties (Bammer 2013).

**Fig. 1:**
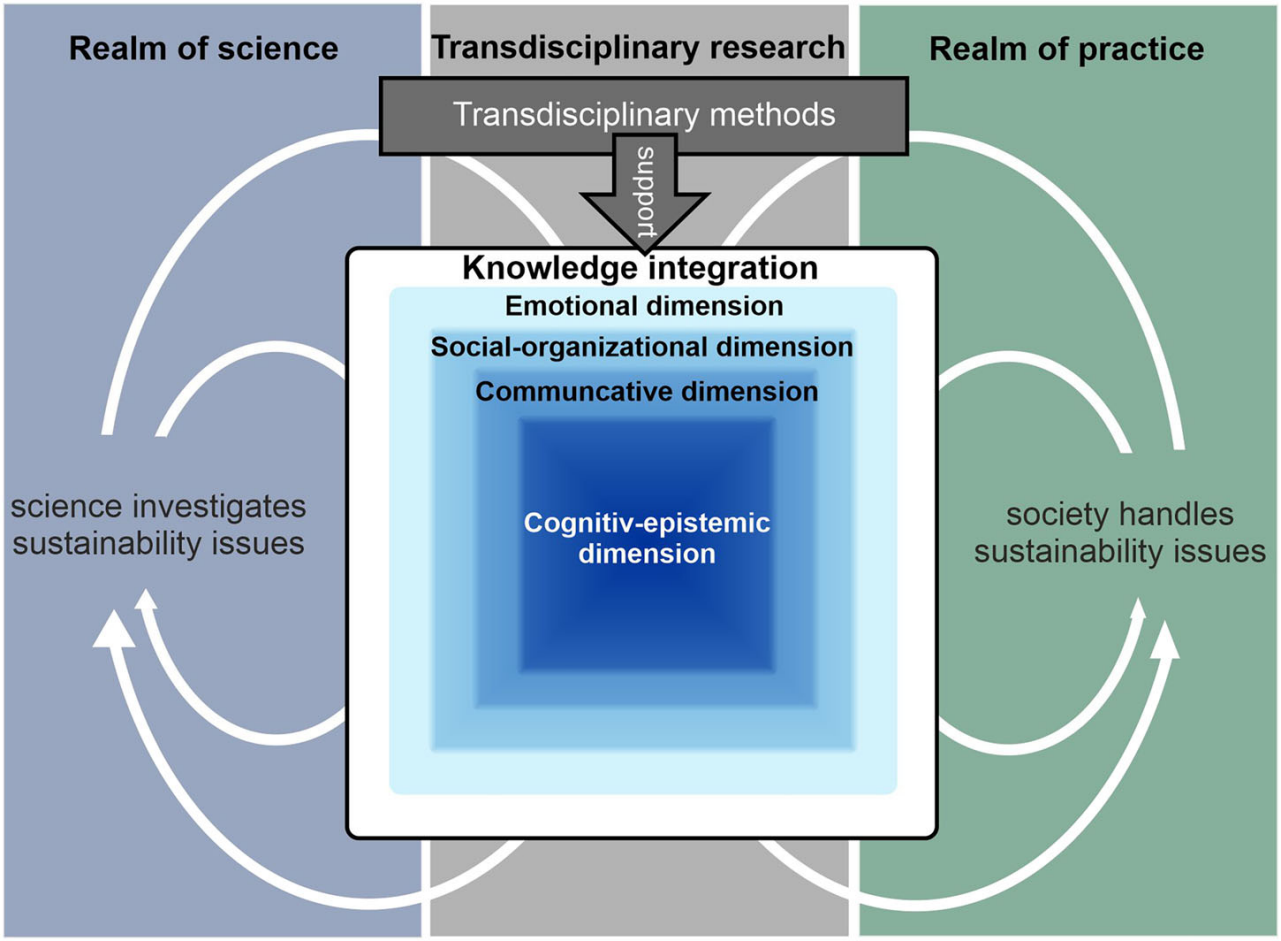
Transdisciplinary methods support knowledge integration across the science-society divide, which encompasses various dimensions, with the cognitive-epistemic dimension at its core. (own illustration, based on Pohl et al. 2017 and Network for Transdisciplinary Research 2024).

Structural, communicative, and value-based barriers hinder knowledge integration, underscoring the need for suitable TdM to effectively organize joint research activities and facilitate the iterative integration of diverse knowledge in td research (Arpin et al. 2023; Misra et al. 2024; Pärli 2023). These TdM need to be continuously developed and adapted through systematization and evaluation to ensure their appropriate selection and effectiveness in specific contexts and for specific purposes (Lam et al. 2021; Pohl et al. 2017) (see Fig. 1).

### Transdisciplinary methods

TdM are structured, reproducible tools that integrate diverse ways of thinking, mitigate power imbalances, and develop problem-oriented solution strategies. They make td processes systematic and transparent while addressing different dimensions of knowledge integration (Becker 2006; Defila and Di Giulio 2018; Jahn et al. 2006; Lam et al. 2021; Lux et al. 2024; Minna et al. 2023; SCNAT Swiss Academy of Sciences 2023). Selecting and adapting TdM requires creativity, reflection, and the ability to transition between different forms of knowledge to align them with the specific research objectives and power dynamics at hand (Bammer 2013; Harris et al. 2024; Minna et al. 2023; Studer and Pohl 2023; Strasser et al. 2014; Theiler et al. 2019). Although TdM specifics are not directly transferable to other projects, their underlying requirements and methodological concepts are. These can be adapted to new scientific and societal problems and their context (Bergmann et al. 2010; Harris et al. 2024; Theiler et al. 2019).

Td research is method-oriented (Andrews et al. 2024; Lang et al. 2012) and requires the selection of an appropriate TdM, a clear purpose for knowledge integration, the identification of common goals and problems, and the development of shared visions to promote social and scientific progress (Defila et al. 2006; Hoffmann 2016; Lang et al. 2012; Misra et al. 2024; Wiek 2007). The diversity of epistemological, ontological, and cultural backgrounds, as well as institutional logics, makes it difficult to determine the best TdM to use (Harris et al. 2024).

The findings indicate that the choice of TdM and the quality of their application have the greatest impact on the success, while institutional factors have a comparatively minor influence (Pärli 2023). The selection and application of methods significantly shape both the outcomes achieved and the knowledge produced (Crawford and Bryce 2003; Pärli 2023).

Td research uses a set of different TdM. The td scenario method, for example, links and synthesizes academic expertise and quantitative system knowledge with normative objectives (target knowledge) and practice-based assessments of transformation pathways by relevant societal actors (Penker and Wytrzens 2005). As a methodological approach to interactive exploration of complex future issues, td scenarios contribute to strategic decision making by co-creating alternative futures (Kerber et al., 2014; McDonald et al. 2009). Another example of TdM is serious games or TdM based on playful elements that can combine cognitive and emotional aspects, foster creative imagination and engagement of actors (Gugerell et al. 2024; Milkoreit 2016; Pärli 2023; Richter et al. 2023; Vervoort et al. 2022). Their playful elements enable social learning and active engagement with complex sustainability issues. By combining scientific and experiential knowledge, they not only promote reflection on complex issues and create innovative spaces for participatory approaches to solutions (Engström and Backlund 2021; Gugerell 2023; Medema et al. 2016; Stacey and Nandhakumar 2009).

### Dimensions of knowledge integration

Scientists often underestimate the requirements for knowledge integration in td research processes by reducing them primarily to the cognitive dimension of knowledge integration (Bergmann et al. 2010), or by using knowledge integration as a ‘buzzword’ to describe a central event within the complex and multifaceted td process (Pohl et al. 2021). For this reason, many authors propose a multidimensional perspective on knowledge integration (Bergmann et al. 2010; Boix Mansilla 2010; Gugerell et al. 2023; Jahn et al. 2012; Mitchell and Ross 2016; Pohl et al. 2021; Pohl 2024), especially when it comes to understanding, monitoring, or managing knowledge integration in practice (Jahn et al. 2012; Pohl et al. 2021). Some authors assume that the cognitive-epistemic dimension of knowledge integration forms the core, though it is supported by other dimensions that play a fundamental role (Boix Mansilla et al. 2016; Scholz and Steiner 2015; Theiler et al. 2019). Td scholars frequently refer to both the concept of knowledge integration and its differentiation into different dimensions of integration. Yet, the number of dimensions involved remains inconclusive and is still debated. The cognitive-epistemic dimension is clearly the conceptual core of knowledge integration and is often defined similarly to knowledge integration itself. Several authors agree, however, that knowledge integration encompasses more than purely cognitive processes (Becker 2006; Bergmann et al. 2010; Bergmann et al. 2010; Jahn et al. 2012; Pohl et al. 2021; Theiler et al. 2019; Scholz et al. 2024a).

In addition to the cognitive-epistemic dimension, which focuses on the creation and evaluation of knowledge, the communicative, social-organizational, and emotional dimensions have been highlighted (Boix Mansilla 2010; Jahn et al. 2012; Mitchell and Ross 2016; Pohl et al. 2021). Considering these different dimensions can improve the knowledge integration process (Strasser et al. 2014), despite their close linkages and mutual dependencies (Becker 2006; Bergmann et al. 2010; Boix Mansilla et al. 2016).

In the *cognitive-epistemic* *dimension*, the aim is to differentiate and link subject-specific disciplinary scientific and experiential knowledge (Enengel et al. 2012). Experiential knowledge stems from personal experience or traditional wisdom. It is often implicit and unstructured. Societal actors hold key knowledge about preferences (target knowledge) and options, as well as barriers to change (transformation knowledge). In contrast, scientific knowledge is explicitly formulated, systematic, and grounded in empirical evidence or established theories (Enengel et al. 2012). Scientific knowledge production is a multidimensional process shaped by methodology, reflection, and sociopolitical influences. Its credibility and impact depend on systematic validation, driving scientific progress and ensuring societal relevance (Durán et al. 2022; Janse van Rensburg and Goede 2019; Zougris 2018). Td research is therefore based on joint learning processes between scientists and social groups (Hirsch Hadorn et al. 2008). Integrating scientific and experiential knowledge requires reflecting on one’s own perspectives and epistemologies, understanding concepts and explanations from other disciplines, recognizing the limitations of one’s own knowledge, and developing methods and theories jointly (Becker 2006; Bergmann et al. 2010; Boix Mansilla et al. 2016; Jahn et al. 2012; Pohl et al. 2021; Sebastián et al. 2025). The cognitive-epistemic dimension involves highly specific learning processes (Burger and Kamber 2003) and requires intellectual openness and a strong willingness to collaborate when dealing with other research practices and ways of thinking (Strasser et al. 2014). Knowledge integration is always recognized as having a cognitive dimension, with cognitive elements acting as both inputs and outputs (concepts, models, data, theories, etc.) (O’Rourke et al. 2016).

The *communicative dimension* focuses on developing a common language that enables effective communication (Bromme 2000; Jahn et al. 2012; Klein 2012; Laursen and O’Rourke 2019; Lux et al. 2024; Peukert and Vilsmaier 2021; Pohl et al. 2021). Language plays a central role in knowledge integration by enabling clear, reciprocal exchanges between different disciplines and actors. In this context, shared terminology and linguistic structures (e.g., isomorphisms and codes) facilitate understanding, promote interdisciplinary dialog, and generate td outcomes (Lux et al. 2024; Pohl et al. 2021; Thompson 2017). Time, opportunities, and support must be provided to establish a common communication practice in everyday research, to clarify different or shared linguistic expressions, meanings, and terms, as well as to develop a standardized language practice or construct new terms (Becker 2006; Bergmann et al. 2010; Fleming et al. 2021; Jahn et al. 2012; Lang et al. 2012). Translating technical terms into everyday language and visualizations can strengthen knowledge integration at the communication level (Fischer et al. 2024; Peukert and Vilsmaier 2021; Theiler et al. 2019). Projects that develop a common language are more likely to succeed and enable new insights into problem solving (Tress et al. 2007; Edmondson and Nembhard 2009).

The *social-organizational dimension* focuses on clarifying and linking the different interests and activities of the scientists and organizational units involved (Jahn et al. 2012). This includes conscious team leadership and a willingness to learn (Becker 2006; Bergmann et al. 2010; Jahn et al. 2012), as well as the search for structural synergies that can be utilized jointly (Peukert and Vilsmaier, 2021). This dimension requires distributing roles and responsibilities, formulating agreements (Strasser et al. 2014; Lux et al. 2024), and managing interdependencies (Bammer 2013). Pohl et al. (2021) refer to this dimension as ‘social-interactive’ and emphasize its growing importance when participants must alter their beliefs.

The *emotional dimension* is strongly characterized by a sense of group identity and participation (Boix Mansilla et al. 2016; Pohl et al. 2021; Fleming et al. 2021). Emotional aspects that support knowledge integration include a sense of belonging, positive emotions toward project members and oneself, respect, admiration and recognition, as well as a climate of sociability, and the pleasure of viewing issues from a new perspective (Boix Mansilla et al. 2016; Fry et al. 2008; Lux et al. 2024; Pohl et al. 2021). Emotional tensions can manifest as stress, frustration, or feelings of competition (Thagard and Kroon 2008 in Boix Mansilla et al. 2016). Fry et al. (2008) recommend promoting interpersonal encounters and mutual appreciation through respectful interactions. They point out that scientists often dismiss these approaches as ‘soft’ and not directly associated with methods that facilitate knowledge integration. Theiler et al. (2019) emphasize that a trusting and understanding work atmosphere positively impacts the quality of collaboration and outcomes in td research. Participants are more likely to identify with the results, use them, and transfer them to other contexts with conviction.

Some authors identify three dimensions of knowledge integration (Boix Mansilla et al. 2016; Theiler et al. 2019), while others propose four (Peukert and Vilsmaier 2021; Pohl et al. 2021). Furthermore, the terminology used to describe these dimensions varies, as does the attribution of certain aspects. The existing literature suggests that additional dimensions may be relevant without providing concrete examples (Pohl et al. 2021). It remains unclear how these dimensions relate to each other or influence the effectiveness of knowledge integration (Boix Mansilla et al. 2016; Pohl et al. 2021). While studies in the td literature often focus on conceptual approaches to knowledge integration or its dimensions, empirically based approaches remain scarce (Peukert and Vilsmaier 2021).

In this paper, we conceptualize knowledge integration as follows (see Fig. 1): Knowledge integration is a multidimensional process, with a cognitive-epistemic dimension core (see Fig. 1). Three additional dimensions—social-organizational, communicative, and emotional—complement and influence this process. Strengthening these additional dimensions provides more favorable conditions for effective knowledge integration.

Td research faces theoretical, empirical, and methodological gaps in knowledge integration. Theoretically, there is a lack of systematic development of integration concepts and a more structured approach to understanding and implementing integration (Pohl et al. 2008, 2021). Empirically, existing evaluation frameworks often fail to assess participant engagement quality and the iterative, reflexive nature of td research (OECD 2020; Restrepo et al. 2018). Methodologically, integration approaches remain underdeveloped and lack clarity and specificity in defining and achieving effective integration (Pohl et al. 2021). Addressing these gaps is crucial for advancing td methods and increasing their effectiveness.

## Methodological approach

The scale was developed as part of the five-year td research project COwLEARNING for sustainable beef and dairy supply in Austria. The project aimed to (i) investigate past changes and sustainability innovations in dairy and beef supply, (ii) evaluate alternative chains for welfare, environmental, and socio-economic aspects, (iii) identify feasible future changes, and (iv) experiment with innovative learning and TdM, including scenario creation and serious game development. Seven natural and social scientists from two Austrian universities (BOKU University and Vetmeduni Vienna) and five cooperation partners (non-governmental organizations) worked together to achieve these goals. Thirty societal actors representing the Austrian beef and dairy supply chain contributed experiential knowledge. They met with the scientists and the cooperation partners in periodic workshops in the td mission arena to identify transition pathways (transformation knowledge) towards a sustainable supply of beef and dairy (target knowledge) (for more details see Frangenheim et al. 2025). In the second year of the project, scenarios of possible desirable futures for sustainable beef and dairy production in Austria in 2050 were jointly created in the td mission arena by linking scientific system knowledge with the experiential target knowledge of the societal actors (Penker and Wytrzens 2005). To explore the One Welfare concept, a serious game was developed in several workshops (Pinillos et al. 2016) by scientists, game designers, cooperation partners, and the societal actors from the td mission arena. The One Welfare concept emphasizes the interconnectedness of animal welfare, human well-being, and environmental health, highlighting that improvements in one domain can positively impact the others (Pinillos et al. 2016).

**Fig. 2:**
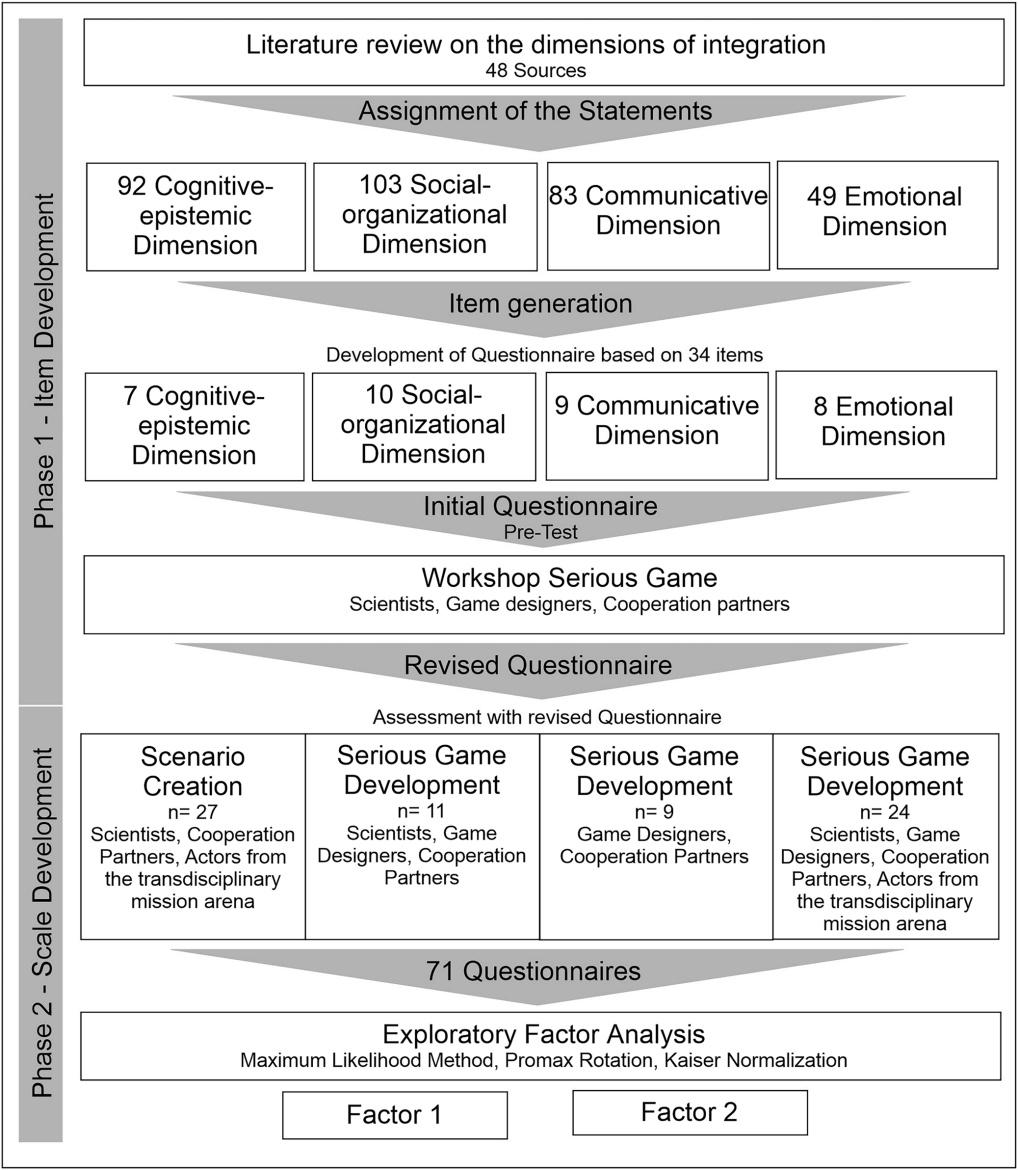
The process of developing a scale to measure the contribution of transdisciplinary methods to knowledge integration, according to Boateng et al. (2018). A statement is a sequence of words that is inductively derived from the literature.

Scales are a way to measure behaviors and attitudes indirectly. They capture complex concepts that cannot be captured by a single variable or item (Boateng et al. 2018). Scales allow for standardized comparisons, tracking of changes, and decision support (Boateng et al. 2018; Morgado et al. 2018). In the first phase, the domain was defined, and the items were generated based on the literature review and feedback from workshop participants. In the second phase, exploratory factor analysis was used to analyze the collected data and construct a scale. In factor analysis, a factor represents an underlying latent construct that accounts for the shared variance among a set of observed items (Eckstein 2016). Building on Fig. 2 and Table 7 in the appendix provides a detailed overview of the steps and methods involved in developing the scale (based on Boateng et al. 2018).

### Phase 1- Item development

To extract the items, a literature review was conducted. On the Scopus and Web of Science databases and with the support of connectedpapers.com, we identified 48 sources that we used to inductively derive 327 statements describing different aspects of integration in interdisciplinary and td research contexts. We condensed these statements into 34 items, which we assigned to the four dimensions of integration (see “Knowledge integration and its dimensions” section). An item is a statement used in a questionnaire designed to capture a respondent’s view. Items are related to a specific concept (Ranganathan and Caduff 2023, compare Fig. 2 and Table 7). Authors who advocated for a multidimensional approach differentiated between three to four dimensions. Depending on the author, some items could have been assigned to different dimensions; thus, the assignment presented in Fig. 2 and Table 3 was unclear.

Next, an initial questionnaire consisting of 34 items across four integration dimensions was developed (see Appendix, Table 6). This initial questionnaire was tested by eleven people in a workshop on creating the serious game. The scientists, game designers, and cooperation partners were asked to check whether the items’ content was clear and comprehensible and whether the items represented the integration dimensions relevantly.

Except for some wording improvements, the participants agreed that the 34 items were appropriate and could be used to measure the contribution of TdM to knowledge integration in td research (see Table 6 in the Appendix). Though some items may have been redundant, the scientists decided not to delete them based on recommendations in the literature (Kline 1993 in Boateng et al. 2018; Schinka et al. 2012 in Boateng et al. 2018). The response option of a continuous scale from ‘strongly agree’ to ‘strongly disagree’ was also tested. Based on the feedback, minor revisions were made, and a revised version of the questionnaire was finalized.

### Phase 2 – Scale development

The revised questionnaire was supplemented with basic background information on the participants, including: Year of birth, gender, level of education, position in the value chain, and zip code. Rather than asking about the four dimensions separately, the items were intermingled in the questionnaire. At the end of each workshop, participants in three game development workshops and one scenario workshop were asked to rate the extent to which each of the 34 statements applied to them. 0.83 percent of the questions were left unanswered and were imputed for further analysis using multiple imputation (Kenward and Carpenter 2007).

### Statistical analysis

Statistical analysis, performed in R, included multiple imputation (Van Buuren and Groothuis-Oudshoorn 2011), descriptive statistics, a correlation structure, and an exploratory factor analysis in order to understand the latent structure of the 34 items (Revelle 2024). The internal consistency of the dataset was evaluated using Cronbach’s alpha and item-total correlations. Cronbach’s alpha evaluates the internal consistency of scale items, i.e., how well the items within a scale correlate with each other. A value of ≥0.70 indicates that the items correlate consistently and are likely to measure the same construct (Bevilacqua et al. 2022; Bonnes and Hochholdinger 2021). On the one hand, while a value of ≥0.90 is considered excellent (Bevilacqua et al. 2022), on the other hand, sources warn that a very high value (>0.90) may indicate that the scale contains too many redundant items, which could limit its usefulness in principal factor extraction (Le Huu Nghia and Thi My Duyen 2019; Tavakol and Dennick 2011). Analyzing inter-item and item-total correlations allows for a detailed examination of the relationships between individual items in an item pool (Boateng et al. 2018). The Kaiser–Meyer–Olkin (KMO) criterion is used to evaluate the suitability of items for extracting principal components. Researchers usually consider a KMO value of 0.7 as an indicator that the items are suitable for the extraction of principal components (Le Huu Nghia and Thi My Duyen 2019). Parallel analysis helps to determine which eigenvalues from the real data are significantly larger than those from comparable random data. Only those factors whose eigenvalues exceed the randomly generated eigenvalues are considered significant (Boateng et al. 2018). The maximum likelihood method was used with an oblique rotation (Promax rotation), as the factors were expected to be correlated, and Kaiser normalization was conducted (Boateng et al. 2018; Bonnes and Hochholdinger 2021; Le Huu Nghia and Thi My Duyen 2019). In factor analysis, a variable’s commonality reflects the proportion of its variance explained by the extracted factors. This indicates how well the factors represent the variable. High commonality means that most of the variance is explained by the factors. Complexity denotes the number of factors needed to explain a variable’s variance. Lower complexity (closer to 1) indicates a clearer fit to a single factor, while higher complexity indicates variance spread across multiple factors. Stevens (2002) recommends interpreting only factor loadings with an absolute value greater than 0.4 (Stevens 2002). Items for which the difference between the primary and secondary loadings is less than 0.2 should not be considered further in the factor analysis (Boateng et al. 2018; Lessiter et al. 2001). Following the factor analysis, factor scores were calculated to quantitatively evaluate the scenario creation and serious game development (Grice 2001).

## Results

The aim was to develop a framework for assessing TdM in supporting knowledge integration within td research by identifying items that can serve as indicators and creating a systematic scale for its evaluation.

### Scale development

The internal consistency of the dataset, as measured by Cronbach’s alpha, was very high across all dimensions and slightly lower within each dimension (see Table 1).

**Table 1:** Internal consistency of the dataset.

Dimension	Cronbach’s alpha (α)
All dimensions	0.95
Cognitive-epistemic dimension	0.82
Social-organizational dimension	0.85
Communicative dimension	0.83
Emotional dimension	0.81

**Fig. 3:**
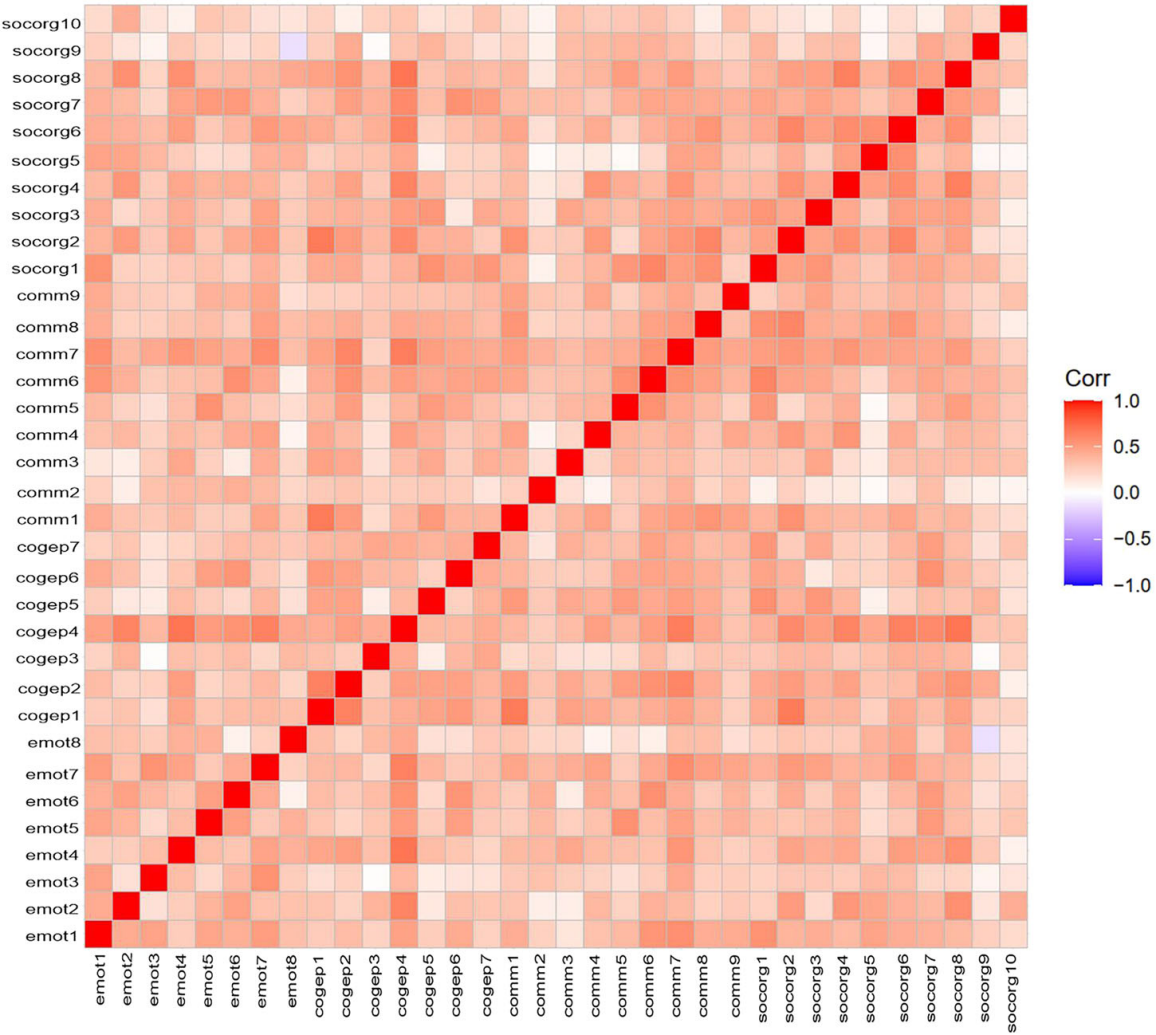
Correlation matrix of all 34 items, which shows similar correlations of all items across all dimensions.

These scores within the dimensions indicated a high degree of internal consistency. The high total score across all dimensions could indicate that more scale items than necessary were developed (Bevilacqua et al. 2022; Le Huu Nghia and Thi My Duyen 2019). This was confirmed by the correlation matrix (see Fig. 3), showing similar correlations of all items across all dimensions, indicating a lack of selectivity between dimensions. The matrix showed that only one correlation was in the negative range (see Table 2). Items such as emot3, emot8, cogep3, comm2, socorg5, socorg9, and socorg10 showed lighter colors, indicating low correlations.

**Table 2:** Results of exploratory factor analysis with two factors.

Rotation Method: Promax with Kaiser normalization. *h*^2^: The communalities of the variables indicate how much of the variance of a variable is explained by the extracted factors. com: The complexity of the variables indicates how many factors are needed to explain the variance of a variable. Fields shaded gray: Items with charge differences ≤0.2 or a load <0.4

**Fig. 4:**
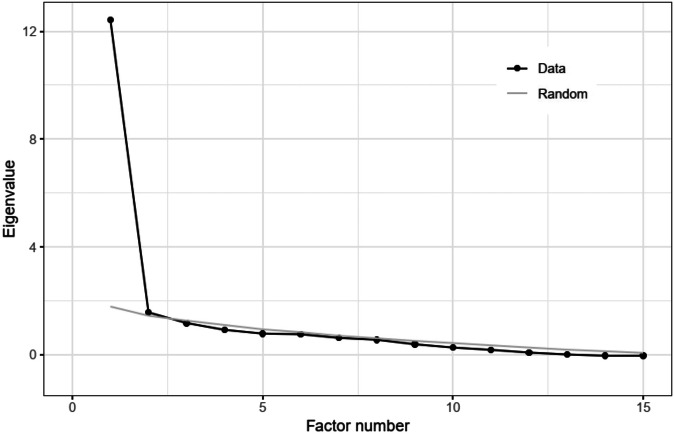
Parallel analysis suggesting two factors, as the eigenvalue for a three-factor model of random data already surpasses the observed eigenvalue for the real data.

The Kaiser–Meyer–Olkin criterion yielded a value of 0.79, confirming the suitability of the items for factor analysis. Although four dimensions were originally derived theoretically, the parallel analysis (see Fig. 4) showed that two factors were statistically significant, and their eigenvalues were higher than those of the randomly generated comparison data. A third factor had an eigenvalue > 1, but this was below the threshold value of the random data and was therefore not considered. This decision followed established methodological recommendations for factor extraction (Boateng et al. 2018).

Exploratory factor analysis (maximum likelihood method) with promax rotation and Kaiser normalization revealed that the 34 items loaded on two main factors (see Table 2), with both factors having similarly significant loadings. The two factors cumulatively explained 42% of the variance, with each factor contributing equally. Factor one explained 48% and factor two explained 52% of the proportion of the total variance. The correlation between the two factors was 0.71.

Table 3 shows the items and the factor loadings for the two factors. It shows that the items of the dimensions ‘emotional’ and ‘communicative’ were perfectly separated after removing overlapping items. The items of the other two dimensions were spread over both factors. A total of 13 items were assigned to factor one and 12 items to factor two. Most of the items, namely five, were removed from the communicative dimension. The item comm2 was not included in the factor diagram due to a loading below 0.4, as well as items with loading differences less than or equal to 0.2 (socorg7, socorg10, comm4, comm7, comm8, comm9, emot1, emot5; see shaded gray fields in Table 2). When considering the two factors together, all items from the cognitive-epistemic dimension, eight items from the social-organizational dimension, four items from the communicative dimension, and six items from the emotional dimension were retained.

**Table 3:** Items of the two factors (ranked by height of the factor loading).

	Item	Shortcut	Factor loading
**Factor 1**	Today’s workshop helped to strengthen our social relationships and to get to know each other.	socorg5	0.9
	Today’s workshop helped me to feel that I and my knowledge are valued.	emot2	0.8
	Today’s workshop helped strengthen my willingness to engage in a joint learning process.	cogep4	0.8
	Today’s workshop helped to strengthen the willingness to work together.	socorg6	0.8
	Today’s workshop helped me feel part of the group.	emot8	0.7
	Today’s workshop helped identify roles, tasks, and responsibilities in the group.	socorg4	0.6
	Today’s workshop helped to support the group in the exchange of ideas and in working together on an equal footing.	socorg8	0.6
	Today’s workshop helped to make the group’s expectations and ideas transparent.	socorg2	0.6
	Today’s workshop helped to combine knowledge from science and practice with the help of the games/scenario development method.	cogep3	0.5
	Today’s workshop provided insights into how regular meetings promote mutual understanding.	emot4	0.5
	Today’s workshop helped to increase my enjoyment of looking at issues from a new perspective.	emot7	0.5
	Today’s workshop helped me to feel safer in the group. It is now easier for me to express doubts or (unusual) ideas.	emot3	0.5
	Today’s workshop helped me to learn more about the milk and beef supply in Austria.	emot6	0.4
**Factor 2**	Today’s workshop provided insight into what knowledge exists in the group, where knowledge overlaps, and what is missing.	cogep5	0.9
	Today’s workshop helped to translate a general question into the specific languages of the participants from science and practice.	comm5	0.8
	Today’s workshop helped to highlight the different activities and interests of the participants.	socorg1	0.7
	Today’s workshop helped clarify terms and their meanings.	comm6	0.7
	Today’s workshop helped to clarify common issues, definitions, and criteria in sustainable dairy and beef supply.	cogep2	0.7
	Today’s workshop provided insight that more coordination is needed when the group composition changes.	socorg9	0.7
	Today’s workshop provided insights into how theoretical models describing complex phenomena or contexts (such as One Welfare, …) are an important means of communication.	comm3	0.6
	Today’s workshop helped to create new knowledge in our group by sharing and linking individual knowledge.	cogep1	0.6
	Today’s workshop helped to achieve a better understanding of the sub-sectors and interfaces of the Austrian dairy and beef supply.	cogep6	0.5
	Today’s workshop helped to bring together the knowledge of those involved through understandable language and terms.	comm1	0.5
	Today’s workshop helped me to better understand how the dairy and beef supply in Austria can be changed and how it can be shaped.	cogep7	0.5
	Today’s workshop helped to reconcile the different activities and interests of the participants.	socorg3	0.5

The items in the first factor focused on promoting the social and emotional dimensions and strengthening cooperation, with the aim of creating a supportive, open, and appreciative environment for knowledge integration. The items of the second factor focused on promoting the cognitive and communicative dimensions, such as the mutual exchange of knowledge within the group, the understanding of complex relationships, and theoretical models.

In the exploratory factor analysis, two factors and their associated knowledge integration items could be empirically distinguished. Considering existing labels and linking the dominant labels identified in the literature, the first factor can be described as the ‘social-emotional dimension’, and the second as the ‘cognitive-communicative’ dimension.

The nine items that did not load onto any factor emphasize the importance of communication in understanding complex issues, reflecting on one’s own work, and collaborating. While the content of the excluded items is important for td collaboration, including them would weaken the power of the scale and reduce the ability to distinguish between the measured dimensions.

### Testing the scale for evaluating serious game development and scenario creation

In the next step, the scale consisting of the items assigned to factors one and two was used to evaluate the contribution of serious game development and the scenario creation to knowledge integration. The average value of the factors was determined and visualized using a box plot comparison to reveal differences in response patterns.

**Fig. 5:**
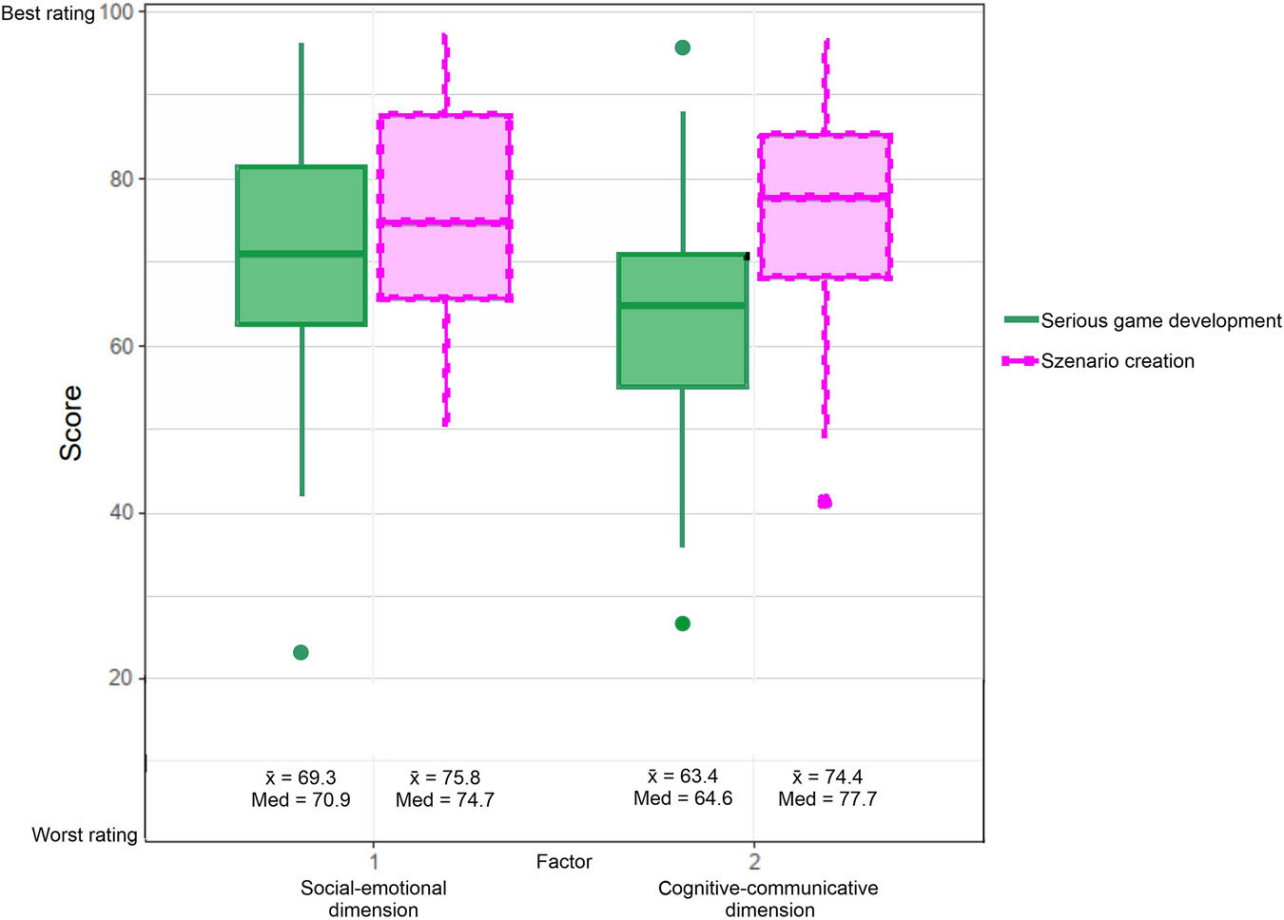
The two transdisciplinary methods are comparatively evaluated by all actors regarding their integration performance (social and scientific actors), which shows that scenario development scores better than serious games creation in both factors. Factor one (mainly emotional and socio-organizational items) has a wider range, while factor two (cognitive and communicative items) shows a statistically significant difference in favor of scenario development.

Figure 5 compares the two TdM based on the aggregated ratings of all actors (the higher the score, the better the rating). The results show that scenario creation achieves higher scores for both factors, as illustrated by the median and the interquartile range. Factor one shows a wider range for both methods than factor two. The mean score for serious games is 69.3 for factor one (mostly emotional and socio-organizational items) and 63.4 for factor two (dominated by cognitive and communicative items). For scenario creation, the mean is 75.8 for factor one and 74.4 for factor two; the median is higher for factor two. The Wilcoxon Rank Sum Test yielded a *p*-value of 0.106 for factor one, while a statistically significant difference with a *p*-value of 0.003 was demonstrated for factor two. It should be noted that the *p*-values are provided for illustrative and interpretive guidance only. The assumption of independence may be violated, as some participants attended both workshops or multiple sessions within one method. This limits the statistical significance of the results, though the values still offer a rough indication of potential differences (see “Limitations” section).

A comparison of the evaluations of the scenarios by the two groups of scientific and societal actors reveals that the societal actors were more positive in their evaluation than the scientific actors (see Table 4). In the development of the serious game, however, the scientific actors rated factor one more positively than the societal actors did, but rated factor two less positively. The evaluation of the scientific actors exhibited a greater degree of variability. Due to the limited sample size, however, the results show a high degree of dispersion, and the group-related analyses lack statistical significance.

**Table 4:** Separate evaluation of TdM by group.

	Factor 1	Factor 2
**Serious game development**		
Scientific actors	x̄ = 70.6, Median = 75.4	x̄ = 58.6, Median = 63.0
Societal actors	x̄ = 68.4, Median = 69.3	x̄ = 66.8, Median = 65.3
**Scenario creation**		
Scientific actors	x̄ = 70.8, Median = 66.5	x̄ = 70.7, Median = 68.1
Societal actors	x̄ = 77.6, Median = 76.0	x̄ = 75.7, Median = 78.5

## Discussion

An increasing amount of td research addresses complex problems by integrating knowledge across the science-society divide. Knowledge integration has been identified as one of the key challenges of td research (Scholz et al. 2024b). Therefore, it is crucial to understand the contribution of TdM to different dimensions of knowledge integration for targeted method selection and development. This exploratory study contributes to the systematic evaluation of TdM by developing and empirically validating a two-factor scale with 25 items to assess its contribution to knowledge integration. Subjective assessments capture aspects of knowledge integration that are not directly observable or objectively measurable. In the absence of objective indicators, using a standardized scale appropriately operationalizes participants’ self-assessments of how they perceive TdM’s contribution to knowledge integration (Defila and Di Giulio 2015). The following section discusses how these findings advance the theoretical and methodological understanding of knowledge integration, explores potential applications of the scale, and addresses its limitations.

### Two factors empirically substantiating the dimensionality of knowledge integration

Exploratory factor analysis revealed two distinct factors, suggesting that evaluating knowledge integration in TdM may be more effective with two factors than with the initially proposed four overlapping dimensions: cognitive-epistemic, social-organizational, communicative, and emotional (Bergmann et al. 2010; Boix Mansilla 2010; Jahn et al. 2012; Mitchell and Ross^ 2016^; Pohl et al. 2021). Parallel analysis supported this finding by showing that the eigenvalue for a three-factor model of random data surpassed that of the real data, justifying the retention of only two factors.

In td literature, models with three, four, or more dimensions are proposed to better understand, analyze, and apply the process of knowledge integration more effectively in practice (Bergmann et al. 2010; Defila and Di Giulio 2019; Pohl et al. 2021; Scholz et al. 2024a; Theiler et al. 2019). While knowledge integration is often conceptualized as a multidimensional process (Pohl et al. 2021), our empirical findings suggest that two overarching dimensions—social-emotional and cognitive-communicative— more effectively capture the core aspects of td knowledge integration than the originally proposed four overlapping dimensions.

Reducing the scale to two factors highlights the need for methodological and theoretical reflection and opens the possibility of refining the items of the scale to deepen the understanding and analysis of the contribution of TdM to knowledge integration. The items representing the two identified factors require further specification, optimization, and empirical testing to provide a clearer and empirically grounded representation of knowledge integration’s dimensionality.

*Factor one*, the social-emotional dimension, emerged in the factor analysis, comprising all items from the emotional dimension, along with five items from the social-organizational dimension and two from the cognitive-epistemic dimension. Items from the emotional dimension reflected shared emotional states and attitudes, such as a sense of belonging, mutual recognition, and collective commitment. Together, these items provided the emotional basis for successful knowledge integration within the group (Boix Mansilla et al. 2016; Pohl et al. 2021; Fleming et al. 2021). While the connection between the cognitive and emotional dimensions is essential for comprehensive knowledge integration, studies show that emotional and creative aspects are often neglected in favor of cognitive approaches and scientific procedures (Gugerell et al. 2024; Pohl et al. 2021). The items of factor one showed how TdM can create a supportive environment, strengthen social relationships, build trust, and foster emotional aspects for a shared learning process. An in-depth study of these items of factor one could further our understanding of the social-emotional support of TdM. Items that loaded particularly strongly on this factor and may be particularly suitable for further research (socorg5, emot2, cogep4, socorg6, emot8) captured key aspects such as a sense of belonging, trust, and willingness to cooperate, which are crucial for the groups functioning (Boix Mansilla et al. 2016; Bromme 2000; Pohl et al. 2021). These items are essential for promoting joint research among different actors and overcoming dissent. Thus, they are crucial for knowledge integration because the process carries risks. When minorities within the group are not adequately included, tensions can escalate, potentially leading to disintegration (Godemann 2008). This underscores the need to evaluate TdM in terms of the social-emotional dimension to ensure an inclusive and effective integration process.

*Factor two*, the cognitive-communicative dimension, encompassed all of the items from the communicative dimension, as well as five items from the cognitive-epistemic dimension and three items from the social-organizational dimension. These items focus on existing or missing knowledge and activities, mutual understanding, and reflexivity. They illustrate how TdM can support developing shared definitions, harmonizing different interests, and achieving a deeper understanding of complex interrelationships (Bergmann et al. 2010; Godemann 2008; Jahn et al. 2012; Strasser et al. 2014; Truffer 2007). Particularly relevant items (cogep5, comm5, socorg1, comm6, cogep2, socorg9) capture how knowledge is identified, shared, and placed in a common context in order to create common ground and close knowledge gaps, and they could be well suited for further research. Factor two emphasizes the central importance of communication in cognitive td processes. Communication plays a critical role in shaping access to information, supporting cognitive processing during the mutual understanding of complex knowledge, and enhancing the recognition of specific contexts (Bergmann et al. 2010; Jahn et al. 2012; Pohl et al. 2012; Strasser et al. 2014). Actors contribute diverse communication practices and thinking styles, requiring a high degree of openness and adaptability (Bergmann et al. 2010; Jahn et al. 2012). TdM can establish a project-specific communication culture by fostering a shared cognitive frame of reference. This is essential for highlighting differences in conceptual meanings and symbol systems and promoting mutual understanding of the joint research (Bromme 2000). TdM’s ability to highlight such differences and build bridges between concepts needs to be examined and evaluated to optimize its contribution to the cognitive-communicative dimension of integration.

**Fig. 6:**
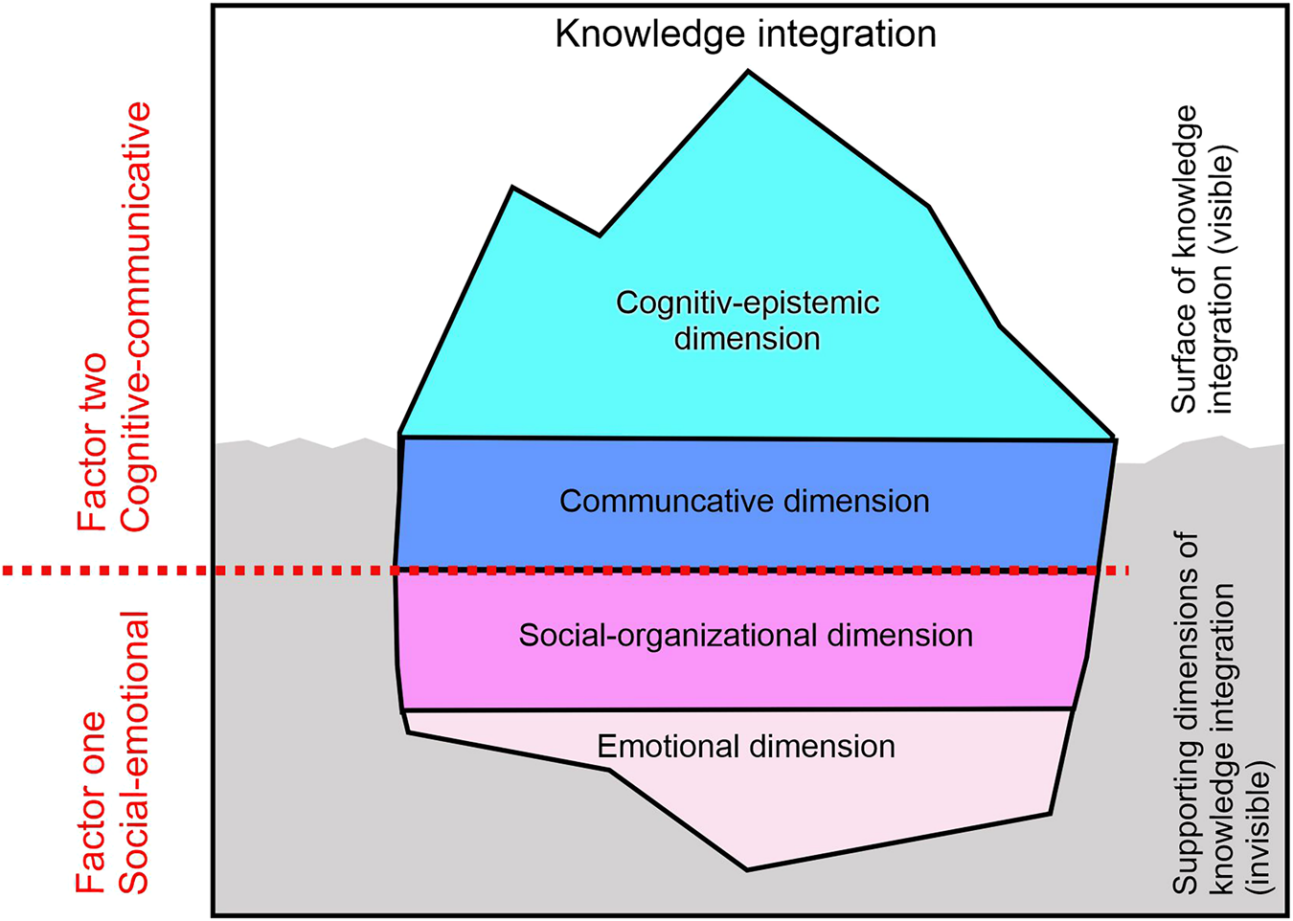
Knowledge integration iceberg model with the two factors for evaluating knowledge integration, with the cognitive-epistemic dimension as the tip of the iceberg (own illustration).

The factor analysis further supports the idea that bridging the gap between science and society requires considering cognitive and non-cognitive aspects of knowledge integration to understand and improve the knowledge integration process (Bergmann et al. 2010; Godemann 2008; Becker 2006; Defila and Di Giulio 2019; Peukert and Vilsmaier 2021; Scholz et al. 2024a). This finding aligns with the literature’s broader call to move beyond the cognitive-epistemic focus that has historically dominated td research (Bergmann et al. 2010; Jahn et al. 2012; Hirsch Hadorn et al. 2008; Pohl et al. 2021). Our knowledge integration iceberg model synthesizes our findings into an additional conceptual model (see Fig. 6), which provides a beneficial conceptualization of the two factors on which future scale development could focus. Based on previous findings, it illustrates that the visible part of factor two is only the tip of the iceberg, while the deeper, invisible layers of factors one and two influence the success or failure of knowledge integration (Fry et al. 2008; Theiler et al. 2019). Considering these findings, any measurement of TdM support for knowledge integration must consider both the visible outcomes and the deeper processes highlighted by the iceberg model in Fig. 6.

In the exploratory factor analysis, two factors could be empirically distinguished, which we have termed the ‘social-emotional dimension’ and the ‘cognitive-communicative dimension’. Alternatively, we could have used new terms such as ‘sense of belonging’ and ‘intellectual discourse’ to emphasize social cohesion and processes of communication and knowledge transfer, respectively. We deliberately avoided new terminology because it could potentially lead to confusion and hinder linking to existing studies. Instead, we drew on established terms from the literature to ensure continuity and traceability, and we encourage future research to further operationalize these two factors.

The items used in this study to measure knowledge integration can be applied to comparable contexts in the Global North. Whether the underlying dimensions remain stable in other contexts and whether the items used in this study will also be assigned to the same factors requires further empirical investigation. Future research in different regional and thematic settings will help to validate the dimensions’ transferability and to identify possible context dependencies or variations in the factor structure.

### Contributions to conceptual clarity

The terms knowledge integration and cognitive-epistemic dimension are widely used in the td discourses, but their demarcation and relationship to each other often remain unclear. Both terms have similar, if not identical, attributes, which leads to notable overlap and ambiguity, creating ‘gray spaces’ that require interpretation, resulting in increased fuzziness (Becker 2006; Bergmann et al. 2010; Godemann 2008). To develop a scale for assessing TdM, we conceptualized knowledge integration as a multidimensional process with a cognitive-epistemic dimension at its core (Fig. 1, presenting the iceberg model of Fig. 6). We emphasize the need for ongoing scientific discourse to further refine and develop the theoretical foundations of a knowledge integration scale.

The literature review revealed conceptual ambiguity in the four dimensions of integration (Boix Mansilla et al. 2016; Theiler et al. 2019; Pohl et al. 2021), which was confirmed by the high overall Cronbach’s alpha value (0.95). This suggested redundancy among the scale items, a lack of distinctiveness between the dimensions, and that they likely measured a similar construct (Le Huu Nghia and Thi My Duyen 2019; Tavakol and Dennick 2011). The correlation matrix further supported these previous findings, showing consistent correlations across all items, indicating that the items did not selectively capture distinct constructs.

The dimensions proposed in the literature provide a structured approach to understanding knowledge integration across contexts. However, their boundaries often blur due to overlapping aspects (Peukert and Vilsmaier 2021; Strasser et al. 2014). For example, the concept of mutual understanding is sometimes assigned to both the communicative and social-organizational dimensions (Bergmann et al. 2010; Pohl et al. 2021). Similarly, the social-interactive aspects of Boix Mansilla et al. (2016), such as group identity or the quality of social interactions, include items that can be found in the emotional dimension of Pohl et al. (2021). These overlaps demonstrate that the theoretical dimensions have not yet been conclusively differentiated. This could be due to the inherent complexity of td processes, in which cognitive, communicative, social, and emotional aspects are often closely interwoven. ‘Diversity of activities and interests’ or ‘group dynamics’ can include organizational, communicative, and cognitive items. Additionally, context-specific application of methods and group work dynamics might shift emphasis from one dimension to another. This leads to additional fuzziness in the delineation of the dimensions (Becker 2006; Bergmann et al. 2010; Boix Mansilla et al. 2016).

This is also corroborated in our study, in which the unexpected loading of cognitive-epistemic items (cogep3, cogep4) on factor one underscores the difficulties in clearly delineating integration dimensions in td research. These challenges warrant further examination in future studies. These items need to be critically reviewed to ensure that they adequately represent the theoretical framework of the dimension. While the other items assigned to factor one mainly emphasized the social and interpersonal aspects of the groups, item cogep3 (‘Today’s workshop helped to combine knowledge from science and practice with the help of the games/scenario development method.’) focused more on linking knowledge. This item could play an important role within the social dimension, as it emphasizes the connections that enhance collaboration and support the integration of scientific and experiential knowledge, thus facilitating knowledge integration. Furthermore, the successful integration of knowledge within the group can strengthen trust and cooperation (Boix Mansilla et al. 2016). On the other hand, item cogep4 (‘Today’s workshop helped strengthen my willingness to engage in a joint learning process.’) could refer more to the willingness to engage in a joint learning process than to the learning itself. In this context, playful elements, such as serious games, can facilitate the integration of cognitive and emotional dimensions, thereby stimulating creative imagination. While the process itself may be the primary focus for some actors, the use of emotionally engaging processes that foster empathy can positively influence the mutual transfer of knowledge (Gugerell et al. 2024; Vervoort et al. 2022).

Item emot6 (‘Today’s workshop helped me to learn more about the milk and beef supply in Austria.’) should also be critically examined in relation to factor one. Although the allocation to the emotional dimension seems inappropriate at first glance, this is explained by the fact that the method was used for knowledge exchange and experience within a group. Sharing information about the dairy and beef supply in Austria fostered a stronger sense of belonging. The common thematic focus may have strengthened knowledge sharing within the group (Boix Mansilla et al. 2016; Henry et al. 1999; Pohl et al. 2021). Nevertheless, the wording of this item requires critical review.

The three items from the social-organizational dimension (socorg1, socorg9, socorg3) warrant a more critical examination in relation to factor two. Items socorg1 and socorg3 (‘Today’s workshop helped to highlight/reconcile the different activities and interests of the participants.’) could emphasize intellectual engagement with the diversity of activities and interests represented in a group, thus loading onto factor two. Item socorg9 (‘Today’s workshop provided insight that more coordination is needed when the group composition changes.’) is highly contextual, as it is only relevant when the composition of the group changes. Such variability may limit the comparability and generalizability of the item across different group constellations. It does indicate organizational and dynamic aspects of group work. Therefore, in further studies, it should be examined whether it would be better to assign it to factor one in further studies—if changing group composition is relevant in these studies at all.

The distribution of items from the social-organizational and cognitive-epistemic dimensions across the two factors underscores the need for more precise item formulations and assignments in future studies. Clearly defining the dimensions and reducing them to two consistent factors could eliminate redundant or unclear items and strengthen the validity of the scales for evaluating TdM. Additionally, context-specific items such as socorg9 should be reevaluated and specified to increase their relevance and comparability in different group constellations. This would promote the comparability among studies and contribute to developing evidence-based recommendations for designing td processes.

Our findings underscore the need for clearer differentiation between cognitive-communicative and social-emotional aspects of knowledge integration in td research. By empirically identifying two distinct factors, we challenge previous models with overlapping dimensions and propose a scale to take a more structured approach to measurement.

### Implications for transdisciplinary research practices

Based on the items assigned to the two factors in Table 3 and the limitations identified (items deemed ambiguous and requiring further research), practical implications can be derived. These implications are listed in Table 5. Only those items for which there is no immediate need for further research were considered. Items requiring more critical review and reflection were deliberately excluded in order to ensure a precise and well-founded derivation of practical applications.

**Table 5:** Practical implications derived from the assigned items.

Factor one: social-emotional dimension	Factor two: cognitive-communicative dimension
Strengthen social relationships and enable mutual learning	Identify what knowledge is available in the group, where knowledge overlaps, and where knowledge gaps may be
Make participants feel that their knowledge is valued	Translate a general question into the specific languages used by those involved
Improvement of cooperation	Clarify terms and their meanings
Strengthen sense of group belonging	Clarify common issues, definitions, and criteria
Identify group roles, tasks, and responsibilities	Use theoretical models that describe complex phenomena or relationships as an important communication tool
Help the group share ideas and work as equals	Share and link individual knowledge
Make transparent what the group expects and ideas	Understand sub-sectors and interface issues
Foster mutual understanding through regular meetings	Use clear language and terminology to reinforce participants’ knowledge
Look at issues from a new point of view	Promote understanding of change
Make the group feel safe	

### Evaluating TdM and their support for knowledge integration using a scale

Applying the scale to the TdM used in the project shows that scenario creation makes a greater contribution to knowledge integration in both factors than serious game development. The scale provides a structured basis for systematically analyzing the effectiveness of different context-specific implementations of TdM and identifying method-specific strengths and weaknesses as well as group-specific differences. The scale supports an evidence-based evaluation of method performance and provides insights into their applicability and acceptance.

At the same time, the scale serves as a tool for reflection, enabling actors to critically assess the contributions of methods to different dimensions of knowledge integration (Boateng et al. 2018; Bammer 2013). This provides the basis for the targeted further development and optimization of methods. Differentiating into two factors – social-emotional and cognitive-communicative – provides a clearer delineation, increasing the validity of the evaluation and facilitating comparability between projects and actor groups.

The scale allows for flexible approaches. Actors can select methods specifically according to dimensions or strengthen weaker aspects in a targeted manner to promote balanced applications. Additionally, it supports critical self-assessment and the adaptation of strategies for knowledge integration within specific research contexts. These insights help to develop empirically based recommendations for designing td processes, improving existing toolkits, and applying them in a more structured way. Integrating the scale into such toolkits enables users the opportunity to select and further develop suitable methods based on empirical evidence of their contribution to integration.

### Limitations

Although the results seem promising for future td processes and td research, there are some limitations to consider. Developing a scale to empirically measure the contribution of TdM to knowledge integration is a complex and time-consuming process (Boateng et al. 2018). While the empirical results are valid and correct, a larger sample is needed to improve the reliability and validity of the scale and to refine and align it with the Boateng et al. (2018) approach. An increased sample size would allow for a more detailed and nuanced analysis of the participating actors, resulting in more precise and differentiated conclusions. In our study, full sample independence could not be ensured, as some individuals participated in multiple workshops while others took part once. Due to the anonymous data, repeated participation could not be tracked, which prevented paired analyses. Consequently, we treated the samples as independent, despite potential dependencies. Future studies should aim for more controlled sample designs with either paired or clearly independent random assignments to maximize statistical validity and test power.

Additionally, the effectiveness of TdM depends heavily on the actors involved and the quality of the implementation of the methods. As the TdM is not directly transferable to other contexts, evaluating TdM scenario creation and serious game development may vary depending on the research context under consideration. However, the underlying requirements and concepts of the methods (Bergmann et al. 2010; Theiler et al. 2019) are transferable. Therefore, evaluating their suitability remains useful for gaining relevant insights and testing their contribution to knowledge integration in different contexts, in terms of both topic and geographic region.

The scale helps capture participants’ subjective perceptions of knowledge integration. It focuses on the process and does not capture the quality or relevance of the generated knowledge. In the future, it could be complemented with other approaches that assess additional aspects of knowledge integration, such as the outcomes and societal impacts of the produced knowledge.

In addition to the previously mentioned potential ambiguities, a response bias in our survey may have affected the quality of the data. Participants showed a general tendency to give either high or low ratings, regardless of the specific content of the questions. This tendency, along with the resulting high correlations, made it difficult to differentiate between items. These limitations may have affected the analyses of scale development. It is important to consider these potential biases to avoid misinterpretation. Future studies should address these issues to ensure further refinement before drawing definitive conclusions.

Exploratory factor analysis provides an initial basis for the further development of a scale to evaluate TdM regarding their contributions to different dimensions of knowledge integration. Since the theoretical dimensions of knowledge integration are difficult to operationalize (Peukert and Vilsmaier 2021), we propose a selection of empirically tested items and recommend refining the scale further to measure the contribution of the TdM to knowledge integration. With these items, two empirically confirmed dimensions of knowledge integration can be distinguished (a social-emotional one and a cognitive-communicative one). Future studies should test the fit of specific items and validate the scale using larger samples to enhance its reliability and accuracy. We do not consider the scale to be exhaustive. It can and should be complemented by other evaluative approaches that address additional aspects of knowledge integration, particularly its quality and societal relevance.

## Conclusions

The article presents the empirical development and application of a scale that systematically measures the contribution of TdM to knowledge integration. This scale enables a statistically substantiated evaluation of TdM and is a useful complement to qualitative evaluation approaches. Exploratory factor analysis revealed two factors representing the dimensionality of knowledge integration: social-emotional and cognitive-communicative. The social-emotional factor encompasses aspects such as trust, belonging, and collaboration. The cognitive-communicative dimension includes mutual understanding, knowledge sharing, and reflexivity. The scale serves as an evaluation and reflection tool for scientific and societal actors as well as facilitators. It supports the systematic evaluation of knowledge integration, evidence-based selection, and targeted adaptation of TdM. Additionally, the scale facilitates comparisons between projects and groups of actors using the same TdM.

The developed scale has several potential applications: (A) It supports the empirically founded selection of TdM in td research by revealing the strengths of methods in specific dimensions. In projects where actors do not know each other, socially-emotionally focused TdM can foster trust and relationship-building, both of which are crucial for effective knowledge integration. As group cohesion increases, cognitive-communicative methods may enhance mutual learning and knowledge integration. (B) The scale provides a sound basis for developing and adapting TdM by identifying gaps and weaknesses in the socio-emotional and cognitive-communicative dimensions of knowledge integration, enabling targeted methods development. Through critical reflection, actors can refine their strategies context-specifically and increase the effectiveness of their TdM. (C) It supports improving and structuring toolkits by providing empirically grounded insights that enable the targeted selection and development of suitable TdM. Integrating it into toolkits can support an evidence-based design of td processes.

The study makes an important contribution to the structured evaluation of TdM; however, further research is needed. Future studies should validate the factor structure in different contexts and refine the scale to improve its precision in evaluating the knowledge integration performance of TdM.

## Data Availability

The datasets generated and used in this study are available under a Creative Commons Attribution License at 10.5281/zenodo.15634304, provided that the authors are properly credited.

## Appendix

Tables 6, 7

**Table 6:** The 34 items of the four dimensions of integration (Items marked with an asterisk have been removed from the scale).

Dimensions grouped items	Shortcut
**Cognitive-epistemic dimension**	
Today’s workshop helped to create new knowledge in our group by sharing and linking individual knowledge.	cogep1
Today’s workshop helped to clarify common issues, definitions, and criteria in sustainable dairy and beef supply.	cogep2
Today’s workshop helped to combine knowledge from science and practice with the help of the games/scenario development method.	cogep3
Today’s workshop helped strengthen my willingness to engage in a joint learning process.	cogep4
Today’s workshop provided insight into what knowledge exists in the group, where knowledge overlaps, and what is missing.	cogep5
Today’s workshop helped to achieve a better understanding of the sub-sectors and interfaces of the Austrian dairy and beef supply.	cogep6
Today’s workshop helped me to better understand how the dairy and beef supply in Austria can be changed and how it can be shaped.	cogep7
**Social-organizational dimension**	
Today’s workshop helped to highlight the different activities and interests of the participants.	socorg1
Today’s workshop helped to make the group’s expectations and ideas transparent.	socorg2
Today’s workshop helped to reconcile the different activities and interests of the participants.	socorg3
Today’s workshop helped identify roles, tasks, and responsibilities in the group.	socorg4
Today’s workshop helped to strengthen our social relationships and to get to know each other.	socorg5
Today’s workshop helped to strengthen the willingness to work together.	socorg6
Today’s workshop helped to achieve a better understanding of those involved in the dairy and beef supply chain and their living conditions.	socorg7*
Today’s workshop helped to support the group in the exchange of ideas and in working together on an equal footing.	socorg8
Today’s workshop provided insight that more coordination is needed when the group composition changes.	socorg9
Today’s workshop provided insight into the fact that it takes time to understand the contributions of actors from other areas.	socorg10*
**Communicative dimension**	
Today’s workshop helped to bring together the knowledge of those involved through understandable language and terms.	comm1
Today’s workshop provided insights into how translating expertise into simple formulations helps to understand the Austrian dairy and beef supply.	comm2*
Today’s workshop provided insights into how theoretical models describing complex phenomena or contexts (such as One Welfare, …) are an important means of communication.	comm3
Today’s workshop revealed that people who can communicate well are helpful in overcoming institutional and language barriers.	comm4*
Today’s workshop helped to translate a general question into the specific languages of the participants from science and practice.	comm5
Today’s workshop helped clarify terms and their meanings.	comm6
Today’s workshop helped to establish that the way the team communicates influences the working atmosphere.	comm7*
Today’s workshop helped to recognize that collegial communication is a prerequisite for effective collaboration.	comm8*
Today’s workshop provided insights into how images or tangible objects can be supporting elements in communication.	comm9*
**Emotional dimension**	
Today’s workshop helped us to better deal with negative emotions (e.g., disagreements, information overload, or excessive demands).	emot1*
Today’s workshop helped me to feel that I and my knowledge are valued.	emot2
Today’s workshop helped me to feel safer in the group. It is now easier for me to express doubts or (unusual) ideas.	emot3
Today’s workshop provided insights into how regular meetings promote mutual understanding.	emot4
Today’s workshop provided insights that have been useful in my previous projects and work. They helped me to evaluate whether we are on the right track in this workshop.	emot5*
Today’s workshop helped me to learn more about the milk and beef supply in Austria.	emot6
Today’s workshop helped to increase my enjoyment of looking at issues from a new perspective.	emot7
Today’s workshop helped me feel part of the group.	emot8

**Table 7:** Overview of steps involved in developing the scale (based on Boateng et al. 2018) and the statistical methods used.

Activity	Purpose	Steps	Statistical method used, including a brief description
Item Development			
Domain identification	Define the domain limits to guide item creation.	Clarify the domain’s intended use	
		Ensure no current tools cover the domain	
		Outline the domain	
		Identify any pre-existing dimensions of integration	
Item Generation	Select suitable statements aligned with the defined domain.	Derive statements inductively from the literature review	
		Assign the statements to the dimension of integration	
Questionnaire Development	Find the right items to adequately measure the area of interest	Condense the statements into items	
Pre-testing of the Initial Questionnaire	Ensuring the items are meaningful	Assess items from societal and non-societal actors for clarity and completeness	
		Assess if items reflect the dimension of integration	
Scale Development			
Assessment with Revised Questionnaire	Gathering Data from different actor groups	Collect data in scenario creation and serious game development	
		Imputation of missing data	Multiple Imputation: Estimates missing values to reduce bias and preserve statistical power (Osborn 2014)
Items Reduction	Find appropriate and parsimonious items	Determine whether items reliably measure the same construct	Cronbach’s Alpha: Evaluates internal consistency of a scale (Cronbach 1951)
		Examine the relationships between individual items in an item pool	Inter-Item (assesses homogeneity among items) and Item-total-Correlations (how well each item correlates with the total scale score) (Morgado et al. 2017; Osborn 2014)
			Correlation Matrix: Foundation for factor or principal component analysis; reveals linear correlations (Janssen and Laatz 2007)
		Evaluate the suitability of items for the extraction of principal components	Kaiser–Meyer–Olkin Criterion: Tests sample adequacy for factor analysis (Kaiser 1974)
Extraction of Factors	Identifying hidden Factors that explain the data patterns	Determine the optimal number of factors	Parallel Analysis: Determines the number of factors to retain by comparing actual eigenvalues with those from random data (Brown 2015)
		Reduce dimensionality in scale development	Exploratory Factor Analysis: Identifies latent structures among items (Osborn 2014) Maximum Likelihood with Promax Rotation: Provides robust factor estimates with Promax allowing correlated factors (Janssen and Laatz 2007) Kaiser Normalization: Standardizes factor loadings (Kaiser 1958)
Testing the Scale			
Evaluate transdisciplinary methods	Comparison of transdisciplinary methods and their contribution to knowledge integration	Reveal possible differences in response patterns	Box Plot: shows the distribution of item responses, especially useful for comparing (multiple) groups
		Provide illustrative and interpretative guidance	Wilcoxon Rank Sum Test: A non-parametric test comparing two independent groups; useful when data are ordinal or non-normally distributed (Janssen and Laatz 2007). Note: Due to anonymous questionnaires, we had to assume independent groups even though dependencies were possible. For recommendations, see “Limitations” section.
